# Supplementing Boar Diet with Nicotinamide Mononucleotide Improves Sperm Quality Probably through the Activation of the SIRT3 Signaling Pathway

**DOI:** 10.3390/antiox13050507

**Published:** 2024-04-24

**Authors:** Haize Zhang, Jiawen Chai, Chaoyue Cao, Xiaolin Wang, Weijun Pang

**Affiliations:** Key Laboratory for Animal Genetics, Breeding and Reproduction of Shaanxi Province, College of Animal Science and Technology, Northwest A&F University, Yangling, Xianyang 712100, China; zhanghz@nwafu.edu.cn (H.Z.); jiawen103@nwafu.edu.cn (J.C.); ccy666@nwafu.edu.cn (C.C.); xlwang@nwafu.edu.cn (X.W.)

**Keywords:** boar, sperm quality, nicotinamide mononucleotide, antioxidation, antiapoptosis, SIRT3

## Abstract

Sperm quality is an important indicator to evaluate the reproduction ability of animals. Nicotinamide mononucleotide (NMN) participates in cell energy metabolism and reduces cell oxidative stress. However, the effect and regulatory mechanism of NMN on porcine sperm quality are still unknown. Here, 32 Landrace boars were randomly assigned to four groups (*n* = 8) and fed with different levels of NMN (0, 8, 16 or 32 mg/kg/d) for 9 weeks, and then serum and semen samples of the boars were collected to investigate the function and molecular mechanism of NMN in sperm quality. The results showed that the dietary NMN supplementation significantly increased sperm volume, density and motility (*p* < 0.05). Interestingly, NMN apparently improved the antioxidative indexes and increased the levels of testosterone (*p* < 0.05) in serum. Furthermore, NMN upregulated the protein levels of sirtuin 3 (SIRT3), antioxidation and oxidative phosphorylation (OXPHOS), but downregulated the protein levels of apoptosis in semen. Mechanically, NMN protected sperm from H_2_O_2_-induced oxidative stress and apoptosis through SIRT3 deacetylation. Importantly, the SIRT3-specific inhibitor 3-TYP attenuated the antioxidation and antiapoptosis of NMN in sperm. Therefore, NMN exerts antioxidation and antiapoptosis to improve boar sperm quality via the SIRT3 signaling pathway. Our findings suggest that NMN is a novel potential boar antioxidative feed additive to produce high-quality porcine semen.

## 1. Introduction

Boars are the breeding stock of the herd and are responsible for providing sperm and producing superior piglets. The genetic quality and fertility of boars directly affect the reproductive efficiency of the herd and the growth performance of the offspring [1]. Therefore, the selection of outstanding boars is crucial for swine production. Artificial insemination is currently the most widely used reproductive technique in the pig industry [2]. Semen quality affects the success rate of artificial insemination [3], and the semen quality of boars is affected by several factors, such as genetic, nutritional, physiological and environmental factors [4,5]. Adverse factors lead to a decrease in sperm viability and sperm count, as well as a decrease in sexual desire, which ultimately leads to the culling of boars [6]. How to maximize their reproductive potential by supplementary nutritional activators is an issue that needs to be addressed.

Sperm are highly susceptible to oxidative stress, which results in reduced quality [7]. Moderate amounts of reactive oxygen species can promote the maturation, capacitation and fertilization processes of sperm, but too much reactive oxygen species can negatively affect sperm, leading to abnormal sperm function or reduced fertilization. The literature has shown that antioxidant supplementation in vitro or in addition to semen diluents in vitro protects sperm from oxidative stress [8,9,10]. However, it may reduce the fertilizing ability of the spermatozoa. Therefore, a proper balance between antioxidants and pro-oxidants is needed to ensure they have a good fertilization capacity. Sperm mitochondria are one of the main sites of reactive oxidative stress (ROS) production [11]. Within the mitochondria, antioxidant proteins include superoxide dismutase (SOD), perforin protein (perfringolysin O), mitochondrial peroxidase (MnSOD) [12] and so on. Balance in the mitochondrial antioxidant system is essential for maintaining sperm quality. Sirtuin 3 (SIRT3), a mitochondria-distributed NAD^+^-dependent deacetylase [13], plays an important role in regulating mitochondrial function and attenuating oxidative stress damage [14]. However, it remains unknown whether SIRT3 has a major role in the mitochondria exerted in boar sperm.

Nicotinamide mononucleotide (NMN) is a natural derivative of B vitamins found in some fruits, vegetables and meats [15], widely noted for its anti-aging and antioxidant functions [16] through its conversion to nicotinamide adenine dinucleotide (NAD^+^) in vivo [17]. NAD^+^ is an essential cofactor for many biological redox processes [18]. It has been reported that the systemic administration of NMN effectively enhances the biosynthesis of NAD^+^ in a variety of tissues and organs, including the liver [19], kidneys [20] and testes [21]. Moreover, NAD^+^ is also a substrate for SIRTs, and NMN affects SIRT3 activity by increasing NAD^+^ levels, mediating cellular energy metabolism and aging. NMN has a variety of beneficial effects on animal reproductive function. Its supplementation has promoted oocyte mass in aged mice [22]; has led to the revitalization of oocyte mass and thus restored fertility in aged animals [23]; has reduced ovarian inflammation in mice fed a high-fat diet (HFD) [24]; and has decreased oxidative stress and apoptosis in the oocytes of mice exposed to butylbenzyl phthalate [25]. Furthermore, NMN exerted its protective effects on the testes of diabetic mice by regulating glycolysis in supporting cells to reduce spermatogenic cell apoptosis [26]. Recently, NMN has been found to attenuate testicular spermatogenic disorders induced by AlCl_3_ exposure through the inhibition of apoptosis, promotion of spermatogonial cell proliferation and activation of the glycolytic pathway [27]. NMN supplementation improved the quality of porcine oocytes under heat stress and mitigated meiotic defects induced by EGBE exposure by restoring the level of NAD and mitochondrial function, thereby eliminating excessive ROS [28]. It has also been shown that NMN rescues the quality of aged oocytes and improves subsequent embryonic development in pigs [29]. But it is not documented if this is the case in normal situations with young healthy animals; the effect and underlying mechanism of NMN on mature sperm are still unclear.

The main purpose of this study was to explore the effect and underlying mechanism of NMN supplementation on porcine sperm quality. Here, we found that NMN supplementation improved the boar semen quality by enhancing the sperm antioxidant and antiapoptotic capacity. Mechanically, NMN increased the sperm ATP level, enhanced antioxidation and inhibited apoptosis through the SIRT3/OXPHOS and SIRT3/Ac-SOD2/Cyt c signaling pathways, respectively. Our findings suggest that 16 mg/kg/d NMN supplementation is a novel potential boar feed additive for producing high-quality semen.

## 2. Materials and Methods

### 2.1. Animal Ethics Statement

All the experimental animals used in this study and the samples needed for the experiment were approved by the Animal Welfare Committee of Northwest A&F University (NWAFU-20220930, Yang Ling, Xianyang, China).

### 2.2. Animals and Experimental Design

**Figure 1:**
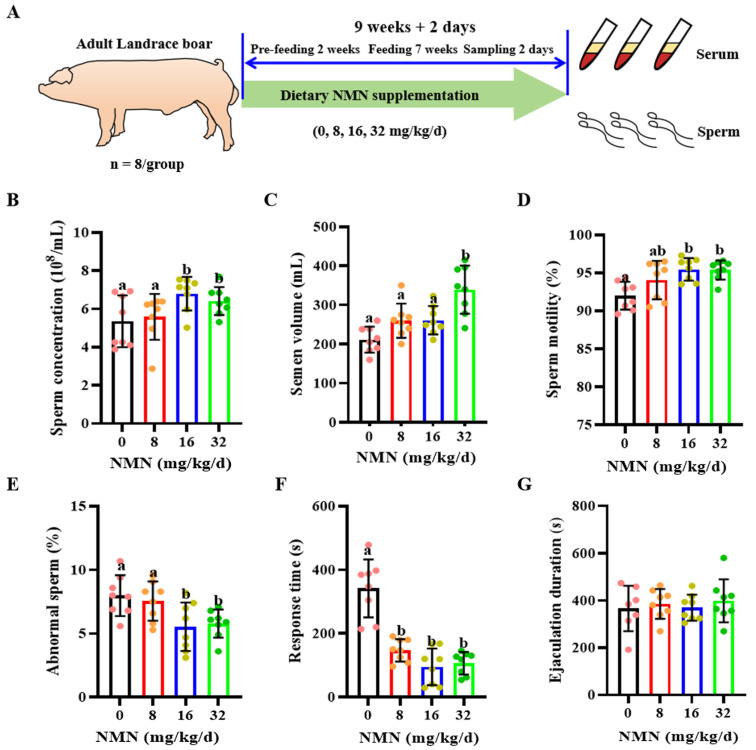
Dietary nicotinamide mononucleotide (NMN) supplementation improves sperm quality and sexual desire in boars. (**A**) The experimental design. (**B**) Sperm concentration. (**C**) Sperm volume. (**D**) Sperm motility. (**E**) Abnormal sperm rate. (**F**) The sexual response time. (**G**) Ejaculation duration. The pink dots indicated the control group, orange dots represented the 8 mg/kg/d group, Yellow-green dots represented 16 mg/kg/d group, green dots represented 32 mg/kg/d. All data are represented as mean ± SEM (*n* = 8 per group). In histograms, different letters indicate significant difference (*p* < 0.05), and no letter or the same letter above the bar indicates no significant difference (*p* > 0.05).

The boars used in this study were obtained from Shaanxi Zhengneng Breeding Pig Genetics Co. in Xianyang, China. According to their weight (250 ± 17.4 kg), 32 Landrace boars were randomly divided into four groups (*n* = 8 per group). The boars were housed in single pens (Figure 1A). The feeding trial lasted for 9 weeks + 2 d, with a two-week pre-feeding phase, a seven-week experimental phase and a 2 d sampling phase. From the pre-feeding phase to the end of the trial, the control (0 mg/kg/d) group was fed with the basal diet, and the treatment groups were fed with a basal diet supplemented with 8, 16 and 32 mg/kg/d NMN, respectively. The NMN supplementation was determined according to boar’s weight and uniformly mixed into their diet. The dosage range of NMN was determined according to the literature in humans [30,31]. The daily feed intake of boars was 2.5 kg/d, and all boars were housed in individual feeding pens (2.2 m × 0.6 m) and fed twice a day (07:00 and 14:00). NMN was obtained from Wuhan Qiancheng Biotechnology Co. Ltd. in Wuhan, China, with purity (HPLC) ≥ 99.0%. Both diets complied with the National Research Council (NRC, 2012) nutritional standards for boars. Diet composition and chemical composition are shown in Supplementary Table S1.

### 2.3. Data Collection and Sampling

Semen and serum were collected from each boar during the sampling period. Briefly, the boars were transferred from the feeding pen to the semen collection pen. The time was recorded that it took for the boar to enter the sperm collection pen to successfully mount the dummy sow stand. The sexual response time was defined as the time from the boar’s entry into the semen collection pen to the time he successfully climbed across the dummy sow stand. The boars’ genital organs and the surrounding area were cleaned with warm water to ensure that the semen collection process was hygienic. Then the boar genitals were stimulated by hand to induce erection and ejaculation. The ejaculated semen was collected into a container using a specialized semen collection apparatus including a filtering device to filter the gel from the porcine semen. During the ejaculation, blood was collected into ethylenediamine tetraacetic acid (EDTA) plasma tubes by venipuncture from the hindlimb vein. Each blood sample was centrifuged at 3000× *g* for 10 min to obtain a serum sample and subsequently stored in liquid nitrogen. The ejaculation duration, semen volume and density were recorded. Ejaculation duration was defined as the time from the start of the boars’ ejaculation to the end of ejaculation. Only one sample (blood and semen) was collected per boar. Sperm quality was detected by a computer-assisted sperm analysis (CASA; Fuzhou Hongye Software Technology Co., Ltd. SAAV6.0, Fuzhou, China). In addition, 10 mL of semen was collected and immediately snap-frozen in liquid nitrogen for the following analyses.

### 2.4. Sperm Motility

Spermatozoa were first centrifuged at 800× *g* for 5 min to remove seminal plasma, followed by re-suspension of spermatozoa using semen diluent and subsequent testing of spermatozoa for various parameters using CASA. A volume of 100 μL of semen sample was placed in a sterilized 1.5 mL centrifuge tube and incubated in a constant-temperature water bath at 37 °C for 5 min. Then, 10 μL of semen sample was taken out and placed on a slide that was pre-warmed in advance, covered with a coverslip and left to stand for a few moments before it could be detected under the CASA microscope. Sperm with a VAP > 5 μm/s were considered as motile. Sperm motility parameters were detected including sperm straight-line velocity (VSL), curvilinear velocity (VCL) and average path velocity (VAP). Five fields of view were randomly selected for measurement, and each field of view contained at least 200 sperm.

### 2.5. Serum Analysis

The levels of NAD^+^, NADH and testosterone were determined in serum using enzyme-linked immunosorbent assay (A&E Bio, Xi’an, China). The levels of NAD^+^, NADH and testosterone were determined by sequentially adding reagents such as samples, standards and horseradish peroxidase (HRP) into the micropores coated with NAD^+^, NADH and testosterone antibody. Serum was processed according to the manufacturer’s instructions and analyzed using an enzyme marker. The levels of ROS (DCFH-DA), MDA (TBA), SOD (WST-1) and T-AOC (FRAP) were detected by the kits (Nanjing Jiancheng Bioengineering Institute, Nanjing, China).

### 2.6. Semen Analysis

Sperm was disrupted using an ultrasonic cell breaker, the supernatant was centrifuged at 1000× *g* for 5 min and the levels of NAD^+^ and NADH were determined (A&E Bio, Xi’an, China). The levels of ROS, T-AOC, SOD and MDA in sperm were detected using the kits (Nanjing Jiancheng Bioengineering Institute, Nanjing, China) according to the previous method [10].

### 2.7. ATP Levels

The ATP levels of sperm were detected using an ATP assay kit (Biotek Biotechnology Co., Ltd., Shanghai, China). First, a standard curve for ATP was prepared by diluting the ATP standard solution on ice to obtain ATP standard solutions. Then the semen samples were washed twice with PBS, and sample concentration was adjusted to 5 × 10^7^/mL. A volume of 200 μL of pre-cooled lysate was added to lysate on ice for 15 min to release intracellular ATP, during which it was broken using an ultrasonic crusher for 30 s. Lysed semen was centrifuged at 12,000× *g* at 4 °C for 10 min, and the supernatant was collected. A volume of 20 μL of the sample was tested and 100 μL of ATP assay working solution were added to a 96-well plate and mixed well. Then, the autofluorescence was measured using a multifunctional enzyme labeling instrument (BioTek, Synergy H1, Winooski, VT, USA). Finally, the ATP concentration of the sample was calculated according to the standard curve.

### 2.8. In Vitro H_2_O_2_ Model Construction

Semen from eight fresh Landrace boars was used as the original semen, and in subsequent experiments, only sperm samples that met the following specific criteria were used: sperm concentration ≥ 5.0 × 10^8^/mL, motile sperm ≥ 80% and normal morphology ≥ 80%. All eligible sperm samples were combined into one sample, taking into account the differences in individual boars. Sperm were diluted to a density of 5 × 10^7^/mL. Beltsville dilution solution (BTS) was prepared by dissolving glucose (37.15 g), EDTA (1.25 g), potassium chloride (0.75 g), disodium citrate (6.00 g) and sodium bicarbonate (1.25 g) in 1 L of double-distilled water. Different concentrations of H_2_O_2_ and NMN were used for incubation at 37 °C for 4 h [32]. The optimal treated concentrations of H_2_O_2_ and NMN were selected for subsequent experiments.

### 2.9. ROS Staining of Live Cells

First, 500 μL of semen was centrifuged at 1000× *g* at room temperature for 5 min, then washed twice with PBS. Then, the sperm were resuspended using 250 mL of 1 μM MitoSOX Red (MCE, Xi’an, China) for ROS detection and 250 mL of 100 nM MitoTracker Green (Invitrogen, Xi’an, China) for labeling sperm mitochondria. Then they were co-incubated for 15 min, washed twice with PBS and photographed under a fluorescence microscope. Finally, the ROS quantification was performed using ImageJ software (v. 1.48).

### 2.10. Sperm Mitochondrial Extraction

A volume of 1.5 mL of semen was centrifuged at 1000× *g* for 5 min, and the sperm were resuspended in PBS and ground 30 to 40 times in an ice bath. Mitochondria were extracted from the isolated sperm using a mitochondrial extraction kit (Solarbio, Beijing, China). The mitochondrial protein extraction reagent A was mixed with mitochondrial protein extraction reagent B, then immediately bathed in ice. DTT was added to a final concentration of 1 mM, and the sperm concentration was adjusted to 2.0 × 10^7^ spz/mL. It was centrifuged (1000× *g*, 4 °C, 10 min) twice to discard the precipitate. The supernatant was transferred to a new EP tube, and the nuclei and unbroken cells were removed. The supernatant in EP tube was centrifuged (10,000× *g*, 20 min, 4 °C), and the supernatant and precipitate were the cytoplasmic and mitochondrial crude products, respectively. The extracted mitochondria were purified by centrifugation (12,000× *g*, 10 min) to remove impurities, and the mitochondrial precipitate was solubilized in Mitochondrial Protein Extraction Reagent C.

### 2.11. Sperm Apoptosis Assay

The early and late apoptosis of sperm were determined using the Sperm Apoptosis Kit Detection (A211-02, Vazyme, Nanjing, China). We stained the cell membrane phosphatidylserine with annexin V marked with biotin and fluorescein isothiocyanate. To prevent experimental errors, the cells were also stained with PI, which stains the DNA. Sperm were analyzed by flow cytometry (BD, Franklin Lake, NJ, USA) [33], and the apoptosis rate was expressed as the proportion of cells in the apoptotic phase. PI-negative and FITC-positive (early apoptosis) as well as PI-positive and FITC-positive (late apoptosis) sperm were considered apoptotic sperm.

### 2.12. Western Blotting

The samples were initially centrifuged at room temperature at 2000× *g* for 3 min. Subsequently, they were washed with PBS and suspended in RIPA buffer containing 1% phenylmethyl sulfonylfluoride (PMSF) and phosphatase inhibitor (HAT, Xi’an, China), as well as 1% protease inhibitor cocktail (EDTA-free, 100 ×; MCE, Xi’an, China) at 4 °C for 10 min. Since the sperm membrane is relatively resistant to disruption, ultrasonication (20 KHz, 750 W, operating at 30% power) was used to lyse the samples in six cycles of 5 s on and 5 s off. After 30 min of lysis at 4 °C, the samples were centrifuged at 12,000× *g* at 4 °C for 10 min. A portion of the supernatant was used to determine the total protein concentration, while the remainder was mixed with 5 × SDS loading buffer and boiled at 95 °C for 5 min. The lysates containing an equivalent amount of protein (30 μg) were analyzed using SDS-PAGE followed by Western blotting, according to a previous study [34]. The PVDF membranes were stripped and then incubated with the primary antibodies overnight at 4 °C. After washing them, membranes were incubated with the secondary antibodies. Both goat anti-rabbit secondary antibody and goat anti-mouse secondary antibody were diluted in 1:10,000. The remaining antibodies are listed in Table 1. Protein quantification was performed using ImageJ software.

### 2.13. Immunofluorescence

According to the standard protocol used in a previous study [35], 10 μL of collected semen was aspirated and uniformly applied to slides. We fixed boar sperm in 4% paraformaldehyde for 30 min and then washed them 3 times (5 min each) with PBS. The spermatozoa were incubated with 2% Triton X-100 for 30 min at room temperature and again they were washed with PBS 3 times. The spermatozoa were blocked with PBS containing 1% BSA and 1% goat serum for 15 min at 17 °C and then incubated with diluted primary antibody (1:150) overnight at 4 °C. The next morning, after being washed three times, slides were conjugated with goat anti-mouse IgG H&L (Alexa Fluor 647) for one hour at room temperature in the dark. We then used DAPI as a nuclear stain and incubated them for 5 min. After washing with PBS, we covered the slides with fade-resistant fixation medium (Vector, Burlingame, CA, USA). The slides were visualized under a fluorescence microscope (Nikon Eclipse C1, Tokyo, Japan) and quantified using ImageJ software.

### 2.14. Statistical Analysis

All data are presented as bar graphs using GraphPad Prism (v8.0.2.263) (GraphPad Software, La Jolla, CA, USA), with each bar representing the mean ± standard error of the mean (SEM), and were analyzed for statistical efficacy by applying the R software package (version 4.0.5). The data were tested for normality, and one-way analysis of variance (ANOVA) combined with Tukey’s test was used to analyze differences between multiple groups using GraphPad Prism. Statistical significance was considered when different letters (a–c) indicated *p* < 0.05.

## 3. Results

### 3.1. Dietary NMN Supplementation Improved Boar Sperm Quality and Sexual Desire

Compared with the control and the 8 mg/kg/d NMN group, the 16 and 32 mg/kg/d NMN groups had significantly increased sperm concentration levels (*p* < 0.05; Figure 1B); the 32 mg/kg/d NMN group had markedly raised semen volume levels (*p* < 0.05; Figure 1C); the 16 mg/kg/d NMN group had improved levels of sperm motility (*p* < 0.05; Figure 1D); and the 16 and 32 mg/kg/d NMN groups had significantly reduced rates of sperm abnormality (*p* < 0.05; Figure 1E). As indicated by Table 2, the 16 and 32 mg/kg/d NMN groups significantly improved the motility performance indexes, including the average path velocity (VAP), curvilinear velocity (VCL) and amplitude of lateral head displacement (ALH) of boar sperm. Moreover, compared with the control, dietary NMN supplementation notably decreased the ejaculatory response time (*p* < 0.05; Figure 1F), but had no significant effect on the ejaculatory duration time (*p* < 0.05; Figure 1G).

### 3.2. Dietary NMN Supplementation Increased Testosterone Levels and Antioxidant Capacity in Boar Serum

**Figure 2:**
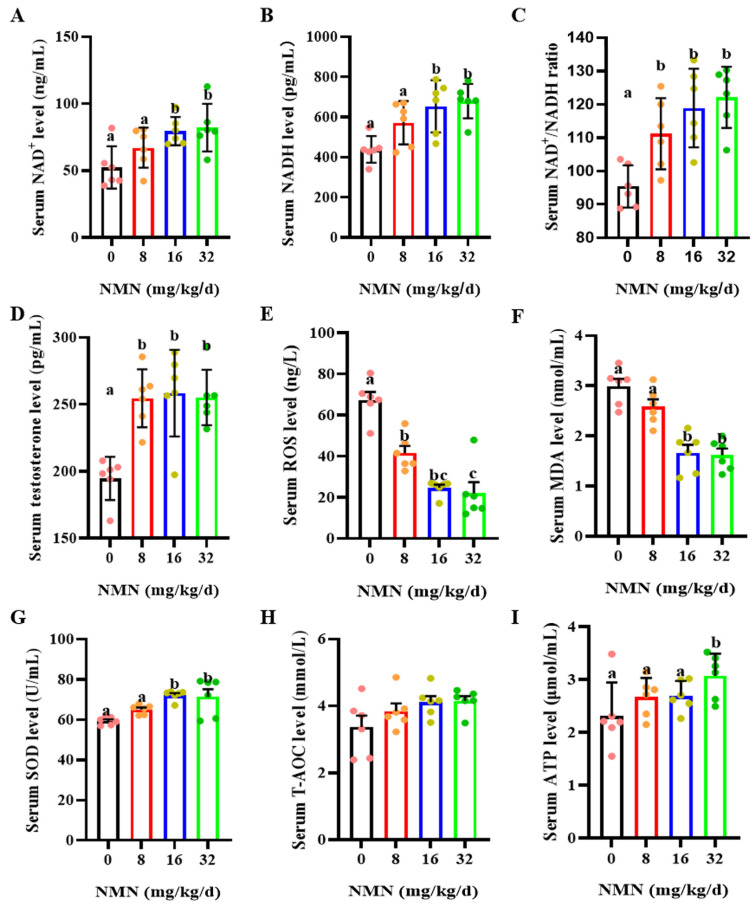
Dietary NMN supplementation increases testosterone levels and antioxidant capacity in boar serum. (**A**) Serum NAD^+^ levels. (**B**) Serum NADH levels. (**C**) Serum NAD^+^/NADH. (**D**) Serum testosterone levels. (**E**) Serum ROS levels. ROS = reactive oxygen species. (**F**) Serum MDA levels. MDA = malondialdehyde. (**G**) Serum SOD levels. SOD = superoxide dismutase. (**H**) Serum T-AOC levels. T-AOC = total antioxidant capacity. (**I**) Serum ATP levels. ATP = Adenosine triphosphate. The pink dots indicated the control group, orange dots represented the 8 mg/kg/d group, Yellow-green dots represented 16 mg/kg/d group, green dots represented 32 mg/kg/d. The data are represented as mean ± SEM (*n* = 5 per group), and different letters indicate significant difference (*p* < 0.05).

As shown in Figure 2, compared with the control, the 8, 16 and 32 mg/kg/d NMN groups had significantly increased levels of serum NAD^+^ (*p* < 0.05; Figure 2A) and NADH (*p* < 0.05; Figure 2B) and an increased serum NAD^+^/NADH ratio (*p* < 0.05; Figure 2C). Additionally, dietary NMN supplementation significantly increased the levels of serum testosterone compared to those of the control group (*p* < 0.05; Figure 2D). Furthermore, compared with the control, NMN supplementation apparently decreased the serum ROS levels (*p* < 0.05; Figure 2E). The 16 and 32 mg/kg/d NMN groups had significantly decreased levels of serum MAD (*p* < 0.05; Figure 2F), but markedly increased serum SOD levels (*p* < 0.05; Figure 2G). NMN supplementation had no effect on the serum T-AOC levels (Figure 2H). Interestingly, the 32 mg/kg/d NMN group significantly promoted the serum ATP levels compared to the other groups (*p* < 0.05; Figure 2I).

### 3.3. Dietary NMN Supplementation Enhanced Antioxidation and Inhibited Apoptosis in Boar Sperm

**Figure 3:**
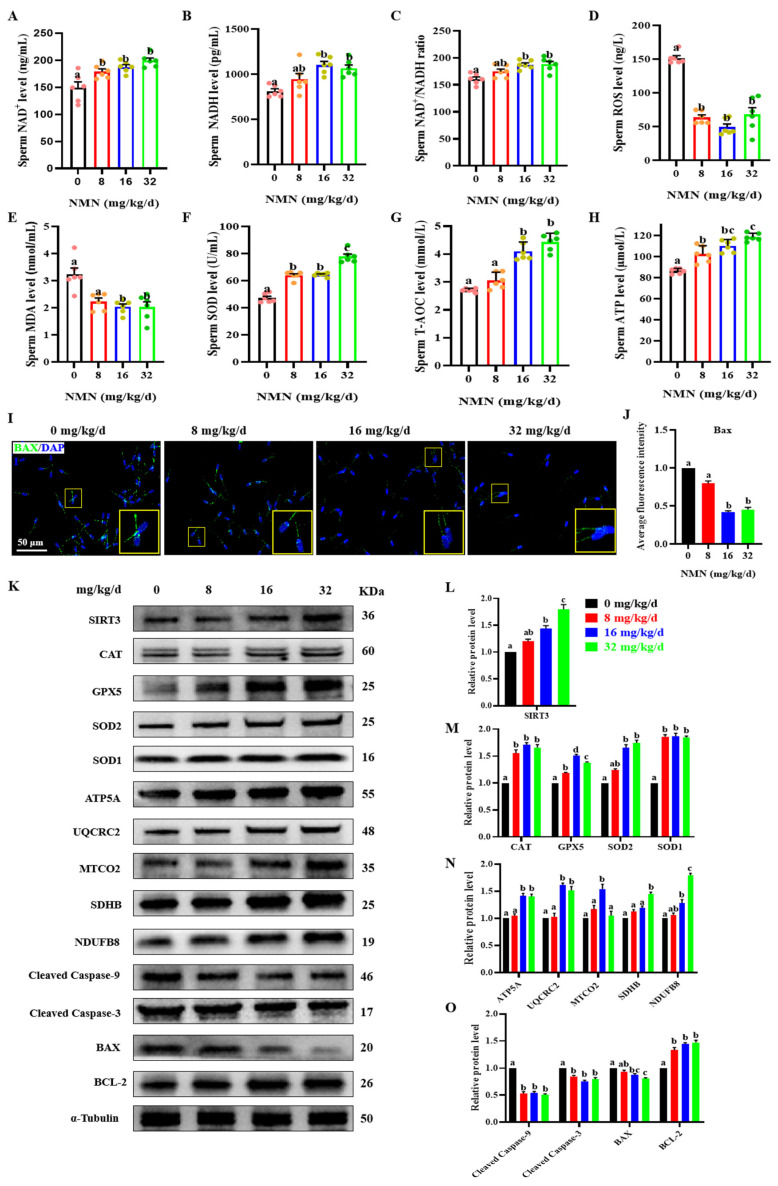
Dietary NMN supplementation reduces ROS and apoptosis levels in boar sperm. (**A**) Sperm NAD^+^ levels. (**B**) Sperm NADH levels. (**C**) Sperm NAD^+^/NADH ratio. (**D**) Sperm ROS levels. (**E**) Sperm MDA levels. (**F**) Sperm SOD levels. (**G**) Sperm T-AOC levels. (**H**) Sperm ATP levels. (**I**) Expression of the apoptotic protein BAX; green = BAX, blue = sperm nucleus. The large yellow pane is an enlargement of the small yellow pane. (**J**) Statistical histograms of sperm BAX green fluorescence intensity. (**K**) Western blot. (**L**) The relative levels of SIRT3 proteins. (**M**) The relative levels of antioxidant proteins CAT, GPX5, SOD2 and SOD1. (**N**) The relative levels of OXPHOS proteins ATP5S, UQCRC2, MTCO2, SDHB and NDUFB8. (**O**) The relative levels of apoptosis-related proteins cleaved caspase-9, cleaved caspase-3, BAX and BCL-2. The samples derive from the parallel experiments and the blots were processed in parallel. The pink dots indicated the control group, orange dots represented the 8 mg/kg/d group, Yellow-green dots represented 16 mg/kg/d group, green dots represented 32 mg/kg/d. The data are represented as mean ± SEM (*n* = 3–5), and different letters indicate significant difference (*p* < 0.05).

To further determine the antiapoptotic and antioxidative effect of dietary NMN supplementation on boar sperm, the levels of sperm oxidative indexes, BAX expression in sperm samples measured by immunofluorescence, and the levels of SIRT3 and key antiapoptotic and antioxidative proteins were detected. As shown in Figure 3, dietary NMN supplementation significantly increased the sperm NAD^+^ level compared to that of the control group (*p* < 0.05; Figure 3A). Compared with the control and 8 mg/kg/d NMN groups, the sperm of groups fed with 16 and 32 mg/kg/d NMN showed an increase in NADH levels (*p* < 0.05; Figure 3B) and NAD^+^/NADH ratios (*p* < 0.05; Figure 3C). In addition, compared with the control group, the NMN groups had significantly decreased levels of sperm ROS (*p* < 0.05; Figure 3D). Feeding the boars 16 and 32 mg/kg/d NMN led to a decrease in the MAD levels (*p* < 0.05; Figure 3E). Feeding the boars NMN increased the SOD level (*p* < 0.05; Figure 3F). The sperm of groups fed 16 and 32 mg/kg/d NMN showed increased T-AOC levels (*p* < 0.05; Figure 3G). Feeding the boars NMN led to increased levels of sperm ATP (*p* < 0.05; Figure 3H). Using the immunofluorescence staining of the apoptotic protein BAX, the sperm of the groups fed 16 and 32 mg/kg/d NMN showed a decrease in the fluorescence intensity of BAX compared with that of the control and the 8 mg/kg/d NMN groups (*p* < 0.05; Figure 3I,J). Furthermore, compared with the control group, feeding the boars 32 mg/kg/d NMN apparently upregulated the protein levels of SIRT3 (*p* < 0.05; Figure 3K,H), as well as antioxidant proteins including CAT, GPX5, SOD2 and SOD1 (*p* < 0.05; Figure 3K,M); promoted the protein levels of oxidative phosphorylation (OXPHOS) including ATP5A, UQCRC2, SDHB and NDUFB8 (*p* < 0.05; Figure 3K,N); and downregulated the protein levels of cleaved caspase-3, cleaved caspase-9 and BAX, but upregulated the protein level of BCL 2 (*p* < 0.05; Figure 3K,O). Overall, these results indicated that dietary NMN supplementation enhanced antioxidation and inhibited apoptosis in the boar sperm.

### 3.4. NMN Protected Sperm from H_2_O_2_-Induced Oxidative Stress and Apoptosis

**Figure 4:**
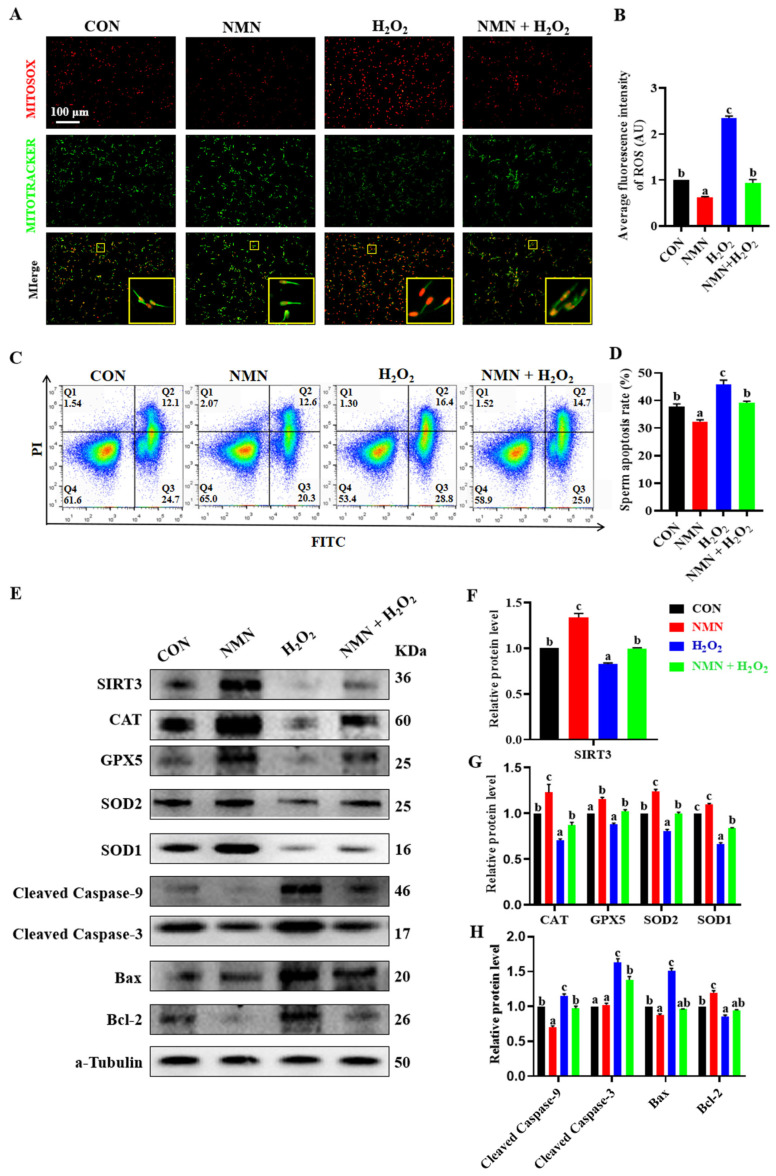
NMN protects sperm from H_2_O_2_-induced oxidative stress and apoptosis. (**A**) Live cell staining of sperm. Red = ROS; green = mitochondria. The large yellow pane is an enlargement of the small yellow pane. (**B**) The fluorescence intensity of ROS. (**C**) Detection of sperm apoptosis by flow cytometry. Q1 is unstained sperm or fragments; Q2 and Q3 are early apoptotic sperm and late apoptotic sperm, respectively; and Q4 is unapoptotic sperm. The blue to red color indicates a gradual increase in the number of cells. (**D**) Apoptosis rate of sperm. Apoptosis rate = early apoptosis rate + late apoptosis rate. (**E**) Western blot. (**F**) The relative levels of SIRT3 proteins. (**G**) The relative levels of antioxidant protein CAT, GPX5, SOD2 and SOD1. (**H**) The relative levels of apoptosis-related protein cleaved caspase-9, cleaved caspase-3, BAX and BCL-2. The samples are derived from the parallel experiments and the blots were processed in parallel. The data are represented as mean ± SEM (*n* = 3), and different letters indicate significant difference (*p* < 0.05).

To confirm the antioxidative and antiapoptotic effect of NMN on porcine sperm, the sperm treated with different concentrations of H_2_O_2_ (0, 50, 100, 150, 200 and 250 μM) and NMN (0, 25, 50 and 100 μg/mL) were used an in vitro model. The results showed that the concentrations of 100 μM H_2_O_2_ and 50 μg/mL NMN were screened according to our evaluation of sperm viability, motility, VAP, VSL and VCL (Figure S1), and the NMN protected the sperm from the decrease in quality caused by the H_2_O_2_ (Figure S2). Furthermore, the NMN apparently alleviated the elevated levels of ROS caused by H_2_O_2_ (*p* < 0.05; Figure 4A,B), as well as significantly inhibited sperm apoptosis induced by H_2_O_2_ (*p* < 0.05; Figure 4C,D). Meanwhile, 50 μg/mL NMN upregulated that the 100 μM H_2_O_2_ induced a decrease in the SIRT3 level (*p* < 0.05; Figure 4E,F), and restored or partially mitigated the protein levels of key oxidant and apoptotic genes, including *CAT*, *GPX5*, *SOD2*, *SOD1*, *Caspase 9*, *Caspase 3*, *BAX* and *BCL 2* (*p* < 0.05; Figure 4E,G,H).

### 3.5. NMN Exerted Antioxidant and Antiapoptotic Effect through SIRT3 Deacetylation

**Figure 5:**
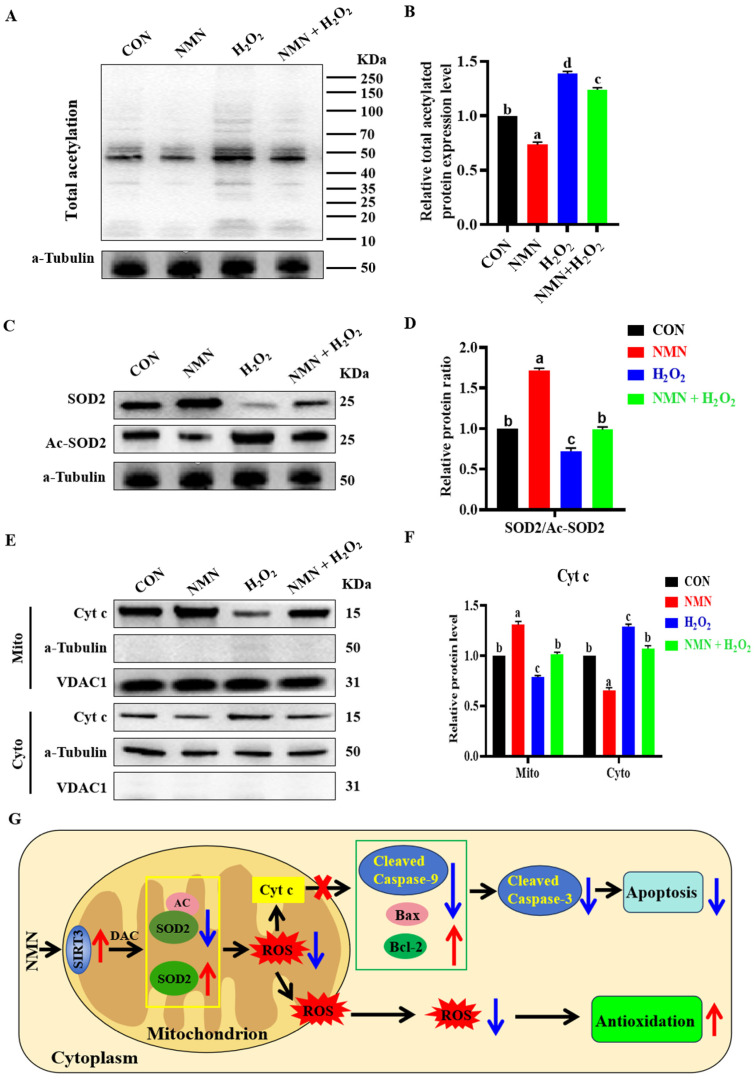
NMN exerts antioxidant and anti-apoptotic effects potentially through activation of SIRT3 deacetylation. (**A**) Detection of total acetylated protein. (**B**) The levels of acetylated protein. (**C**) Western blot for SOD2 and SOD2-acetyl (Ac-SOD2) proteins. (**D**) The relative levels of SOD2 and Ac-SOD2. (**E**) Western blot of Cyt c in mitochondria and cytoplasm. (**F**) The relative levels of Cyt c. (**G**) Schematic diagram of NMN’s role. Red arrows indicate elevated, blue arrows indicate lowered. The samples are derived from parallel experiments and the blots were processed in parallel. The data are represented as mean ± SEM (*n* = 3), and different letters indicate significant difference (*p* < 0.05).

To explore the molecular mechanism of the antioxidation and antiapoptotic effect of NMN on porcine sperm, we detected the levels of total protein acetylation in sperm and the levels of Cytochrome c (Cyt c) in the sperm mitochondria and cytoplasm. The results indicated that 50 μg/mL NMN partially reduced the 100 μM H_2_O_2_-induced increase in total protein acetylation levels (*p* < 0.05; Figure 5A,B). Furthermore, NMN decreased the acetylation level of SOD2 and reduced the regulatory SOD2 acetylation effect of H_2_O_2_ on the sperm (*p* < 0.05; Figure 5C,D). Meanwhile, NMN apparently reduced the level of Cyt-c release from the mitochondria induced by H_2_O_2_ (*p* < 0.05; Figure 5E,F). Therefore, as shown in Figure 5G, NMN had an antiapoptotic effect through the SIRT3/Cyt c/caspase-3 signaling pathway and led to antioxidation by reducing the level of ROS via the SIRT3/AC-SOD2/SOD2 signaling pathway.

### 3.6. SIRT3-Specific Inhibitor 3-TYP Attenuated Antioxidation and Antiapoptotic Effect of NMN in Sperm

**Figure 6:**
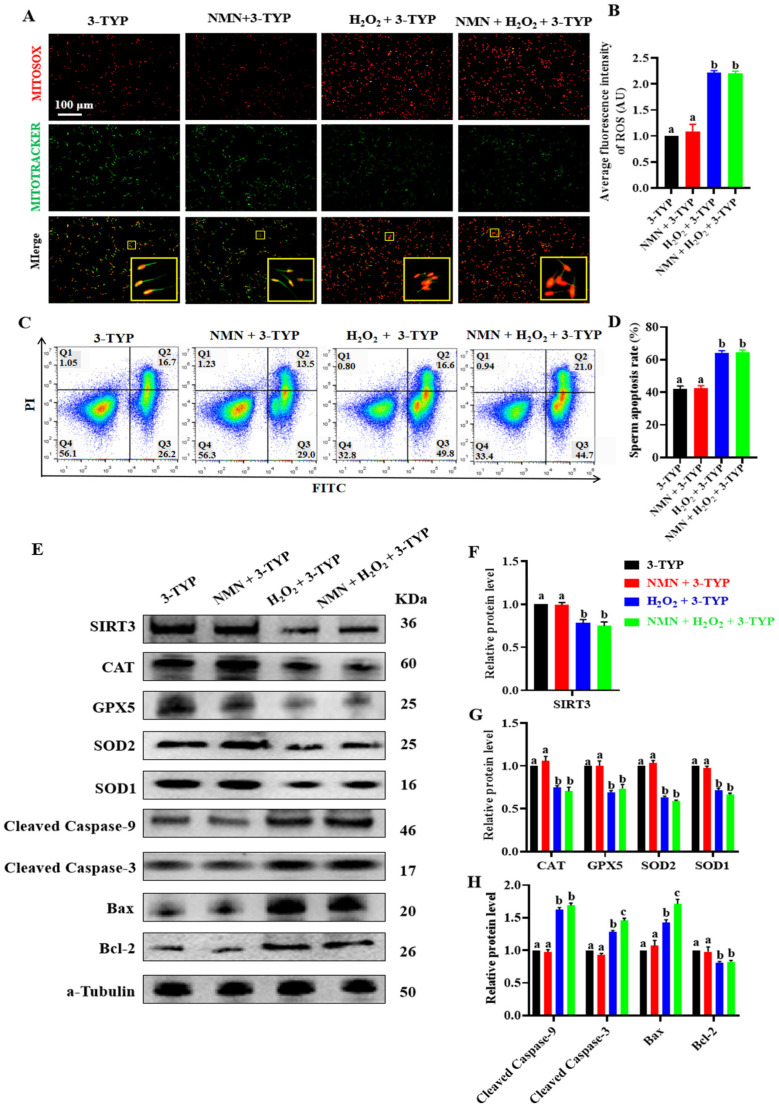
NMN does not exert antioxidant and anti-apoptotic effects in the sperm treated with SIRT3 inhibitor 3-TYP. (**A**) Sperm ROS staining. Red = ROS, green = mitochondria, the yellow pane shows an enlargement of the small yellow pane. (**B**) The fluorescence intensity of ROS. (**C**) Sperm apoptosis assay. (**D**) Sperm apoptosis rate. (**E**) Western blot for the key oxidative and apoptotic proteins. (**F**) The protein levels of SIRT3. (**G**) The protein levels of CAT, GPS5, SOD2 and SPD1. (**H**) The protein levels of cleaved caspase-9, cleaved caspase-3, BAX and BCL-2. The samples are derived from parallel experiments and the blots were processed in parallel. The data are represented as mean ± SEM (*n* = 3), and different letters indicate significant difference (*p* < 0.05).

To show the vital effect of NMN on SIRT3, an experiment was performed using sperm treated with NMN + H_2_O_2_ + 3-TYP (an SIRT3-specific inhibitor). The results showed that NMN failed to alleviate an increase in the ROS levels caused by H_2_O_2_ (*p* > 0.05; Figure 6A,B) and was also unable to attenuate the H_2_O_2_-induced sperm apoptosis when the sperm were treated with 3-TYP (*p* > 0.05; Figure 6C,D). Furthermore, NMN did not change the levels of SIRT3 proteins, antioxidant proteins (CAT, GPX5, SOD2 and SOD1) and antiapoptotic proteins (cleaved caspase-9, cleaved caspase-3, BAX and BCL-2) in the porcine sperm treated with H_2_O_2_ and/or 3-TYP (*p* > 0.05; Figure 6E–H), indicating that the SIRT3-specific inhibitor 3-TYP attenuated the antioxidation and antiapoptotic effect of NMN in the sperm.

**Figure 7:**
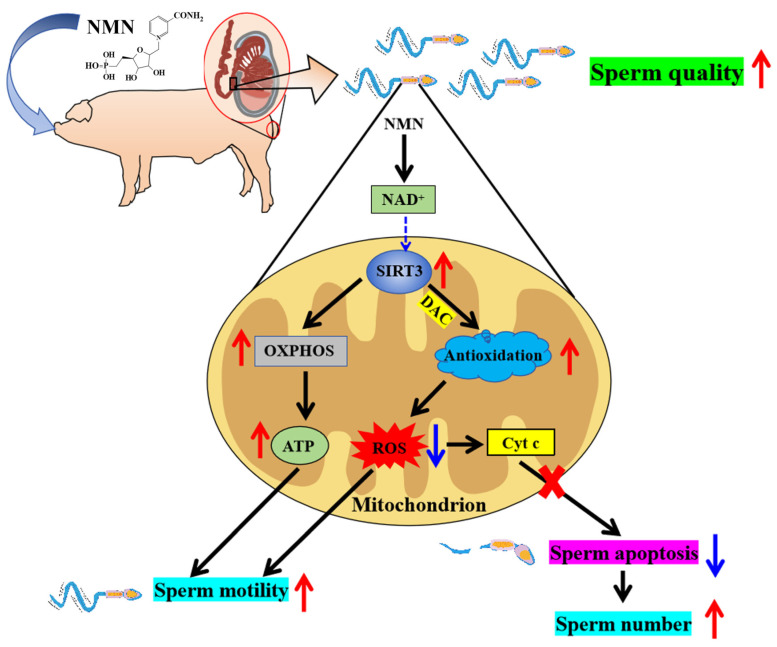
Schematic illustration of the pathways via which dietary supplementation improves sperm quality. As shown in the figure, NMN improves sperm quality by enhancing its antioxidant capacity and inhibiting apoptosis potentially via SIRT3 signaling pathway. Red arrows indicate elevated, blue arrows indicate lowered.

Collectively, NMN is a novel potential boar feed additive because dietary NMN supplementation was shown to improve boar sperm quality through antioxidation, OXPHOS and antiapoptotic effects via the SIRT3 signaling pathway (Figure 7).

## 4. Discussion

In swine production, the most important quality of boars is the provision of superior semen. Dietary nutritional activator supplementation directly and quickly improves the production and quality of boar semen. It was reported that NMN increased the sperm count and reduced sperm deformity in diabetic mice [36]. Moreover, NMN attenuated AlCl-induced testicular spermatogenesis disorders and inhibited spermatogonial cell apoptosis but promoted proliferation [27]. However, there is no literature available to indicate whether NMN supplementation in the spermatozoa of normal young males can exert a beneficial effect. In this study, we have found for the first time that dietary NMN supplementation improves porcine semen quality by increasing ATP levels, enhancing antioxidation and antiapoptotic effects via the SIRT3 signaling pathway.

Mature sperm are highly susceptible to oxidative stress due to the loss of most organelles and cytoplasm except for the mitochondria [11], causing DNA damage, the lipid peroxidation of membranes and damage to the mitochondria, which are all very detrimental to sperm morphology, viability and function [37]. In addition, oxidative stress affects OXPHOS-mediated sperm energy supply, leading to reduced sperm quality [38]. Previous studies have reported that NMN supplementation decreases ROS levels by inhibiting CD38 expression [26], recruits glutathione to repress skin oxidative damage [39] and improves the symptoms of atopic dermatitis in mice by blocking ROS [40]. In the present study, NMN protected sperm from oxidative stress damage by activating SIRT3, which inhibited intracellular oxidative stress. Interestingly, we found that NAD^+^ activated SIRT3 to deacetylate the antioxidant protein SOD2, leading to reduced ROS levels in sperm cells.

It is well known that the ATP level determines sperm motility. There are two pathways for ATP production in sperm for their energy supply, in which the mitochondrial OXPHOS pathway is an important one [41]. Sperm motion (following in a straight line) and speed are important indicators of sperm motility. Previous studies have found that NMN restored the mitochondrial oxidative phosphorylation activity in mouse skeletal muscle [42]. As a therapeutic strategy, NMN improved OXPHOS’s ability to alleviate mitochondrial dysfunction [43]. In this study, we found that dietary NMN supplementation increased the ATP level and motility of sperm by upregulating the levels of OXPHOS. This may be due to the fact that NMN is converted into NAD+ in vivo and participates in redox reactions by activating SIRT3, enhancing the energy supply for sperm respiration [44]. Moreover, NAD+ participated in the reaction between electron transfer chains and provided OXPHOS with the energy needed by cells [45]. Therefore, it makes sense that dietary NMN supplementation improves sperm motility by increasing the sperm ATP level via the SIRT3/OXPHOS signaling pathway.

Sperm apoptosis is one of the most important causes of reduced porcine sperm density and is an important marker of production efficiency. A low rate of sperm apoptosis is indicative of a healthy male reproductive system [46]. There are various factors that lead to sperm apoptosis, such as high temperatures [47], poor feed quality [48] and the frequency of semen collection [49], which severely constrains the efficiency of swine production. NMN rescued the quality of porcine aged oocytes and improved embryo development [50]; it protected corneal endothelial cells from UVB-induced apoptosis [51]. Here, we demonstrated that dietary NMN supplementation increased porcine sperm density by reducing sperm apoptosis. Furthermore, sperm apoptosis is mainly triggered by mitochondria, and the release of Cyt-c is important for initiating the apoptotic cascade response. Cyt-c is an important mitochondrial protein located in the interior of the mitochondria under normal conditions [52]. The oxidative-stress-induced mitochondrial dysfunction caused the release of Cyt-c from the mitochondria into the cytoplasm [53]. Cyt-c in the cytoplasm forms a complex with other proteins known as the apoptosome. It activates caspase-9 and caspase-3, ultimately leading to apoptosis [54]. In this study, we found that SIRT3 suppressed sperm apoptosis by reducing the release of Cyt-c from the mitochondria to the cytoplasm. Therefore, it is very possible that sperm apoptosis is one of the important factors affecting sperm count. Moreover, dietary NMN supplementation increased the porcine sperm count by inhibiting spermatocyte apoptosis and improving proliferation. To sum up, our results suggest that NMN increases the porcine sperm count through inhibiting sperm apoptosis via the SIRT3/Cyt-c/caspase-3 and -9 signaling pathways.

Interestingly, our results found that NMN increased the total testosterone levels and ejaculation duration and decreased the ejaculation reaction time in the boars. Testosterone plays an important role in male reproductive performance [55]. Higher levels of testosterone are usually associated with better sperm quality and quantity, and changes in testosterone levels may also affect the libido and reproductive behavior of boars [56]. We hypothesized that NMN may increase sexual arousal and decrease the ejaculatory response time in boars by increasing their testosterone levels and might increase their sperm count and thus the ejaculation duration by promoting spermatogenesis. However, how NMN affects hormonal changes in boars is still unknown, and further studies are needed.

In this study, no experiments were conducted on the fertilizing ability of the sperm collected from the boars after the supplementation. However, according to previous studies, improving semen quality positively affects their in vitro and in vivo fertilization ability or reproductive performance. For example, the addition of hydroxytyrosol to a dilution of boar semen improved the artificial insemination efficiency by improving the sperm quality [57]. Moreover, the addition of tretinoin enhanced IVF parameters and sperm-oocyte binding by improving the boar sperm quality, increasing the cleavage and blastocyst rates [9]. Therefore, we speculate that NMN may indirectly improve sow fertility and reproductive performance by improving the quality of boar spermatozoa. In order to more comprehensively assess the effects of NMN on boar sperm quality and reproductive performance, future studies could consider in vitro or in vivo fertilization assays to validate our speculations. This would contribute to a more comprehensive understanding of the effects of exogenous supplementation with NMN on boars’ fertilization ability and reproductive performance while improving their sperm quality. Moreover, before adding NMN supplementation to boar diets, it is necessary to assess the fertilizing capacity of their semen, which ensures a thorough understanding of sperm quality and function prior to conducting experiments.

The long and short of it is, we found that NMN not only enhanced boar sexual desire, but also improved the semen quality. Furthermore, because NMN is a natural substance with high safety rating, it might not cause adverse effects in boars. Here, we have for the first time explored the beneficial effects and underlying mechanism of NMN on boar semen quality, and our findings provide an important experimental support for the development of NMN as a boar feed additive.

## 5. Conclusions

In conclusion, dietary 16 mg/kg/d NMN supplementation improved the boars’ semen quality. Mechanically, NMN enhanced sperm viability by activating SIRT3 to upregulate the levels of OXPHOS and antioxidant proteins for higher ATP production and lower ROS levels, and it increased their sperm count by inhibiting sperm apoptosis via the SIRT3/Cyt c/caspase-3 and -9 signaling pathways. Based on these findings, we suggest that NMN could be a novel potential boar feed additive for obtaining high-quality semen.

## Figures and Tables

**Table 1:** The antibodies used in this study.

Antibody	Dilution	Cat. No.	Company
SIRT3	1:1000	AY4134	Abways (Shanghai, China)
SOD2	1:2000	66474-1-lg	Preintech (Wuhan, China)
SOD2 (acetyl K68)	1:1000	CY10546	Abways
CAT	1:1000	21260-1-AP	Proteintech
SOD1	1:2000	10269-1-AP	Proteintech
GPX5	1:600	18731-1-AP	Proteintech
ATP5A	1:1000	CY6775	Abways
UQCRC2	1:1000	CY7110	Abways
MTCO2	1:1000	CY5717	Abways
SDHB	1:1000	CY6860	Abways
NDUFB8	1:1000	CY8290	Abways
Cleaved Caspase-9	1:1000	CY5682	Abways
Cleaved Caspase-3	1:1000	AY0458	Abcam
BCL-2	1:1000	CY5032	Abways
BAX	1:1000	AB3280	Abways
Cty c	1:1000	CY5628	Abways
OPA1	1:1000	CY7035	Abways
Acetylated-Lysine	1:1000	#DF7729	Afinity (Cincinnati, OH, USA)
VDAC1	1:1000	VY5416	Abways
α-TUBULIN	1:1000	AB0049	Abways

**Table 2:** Effect of different NMN supplements on porcine sperm movement parameters.

Item	0 mg/kg/d	8 mg/kg/d	16 mg/kg/d	32 mg/kg/d
VAP (μm/s)	52.33 ± 3.43 ^a^	67.85 ± 1.36 ^b^	68.22 ± 1.52 ^b^	64.73 ± 2.52 ^b^
VSL (μm/s)	32.38 ± 1.91	38.84 ± 3.18	33.38 ± 1.38	31.54 ± 1.24
VCL (μm/s)	105.18 ± 8.46 ^a^	139.80 ± 4.46 ^b^	144.15 ± 5.82 ^b^	144.04 ± 7.53 ^b^
STR (μm/s)	61.90 ±1.21 ^a^	58.62 ± 4.04 ^a^	49.89 ± 2.49 ^b^	51.35 ± 1.76 ^b^
LIN (μm/s)	32.72 ± 1.37 ^a^	30.44 ± 2.92 ^a^	25.72 ± 1.86 ^b^	24.71 ± 0.92 ^a^
ALH (μm/s)	5.81 ± 0.31 ^a^	7.79 ± 0.37 ^b^	7.63 ± 0.30 ^b^	8.29 ± 0.43 ^b^

VAP = Average path velocity; VSL = Straight-line velocity; VCL = Curvilinear velocity; STR = Sperm total motility rate; LIN = Linear motility; ALH = Amplitude of lateral head displacement. ^a,b^ Different letters indicate significant difference (*p* < 0.05).

## Data Availability

The original contributions presented in the study are included in the article/Supplementary Materials, further inquiries can be directed to the corresponding author.

## Supplementary Materials

The following supporting information can be downloaded at: https://www.mdpi.com/article/10.3390/antiox13050507/s1, Figure S1: The optimum treated concentrations of H_2_O_2_ and NMN; Figure S2: NMN protected sperm from decreased motility in sperm treated with H_2_O_2_. Table S1: Composition and nutrient analysis of basal diet.
