# Supplementary material for: Supplementing Boar Diet with Nicotinamide Mononucleotide Improves Sperm Quality Probably through the Activation of the SIRT3 Signaling Pathway
Source: Antioxidants (Basel). 2024 Apr 24;13(5):507. doi: 10.3390/antiox13050507 (PMC11117624; doi:10.3390/antiox13050507)
Supplement: Supplementary file 1 [file antioxidants-13-00507-s001.zip › antioxidants-2968970-supplementary.pdf]

Supplementary File

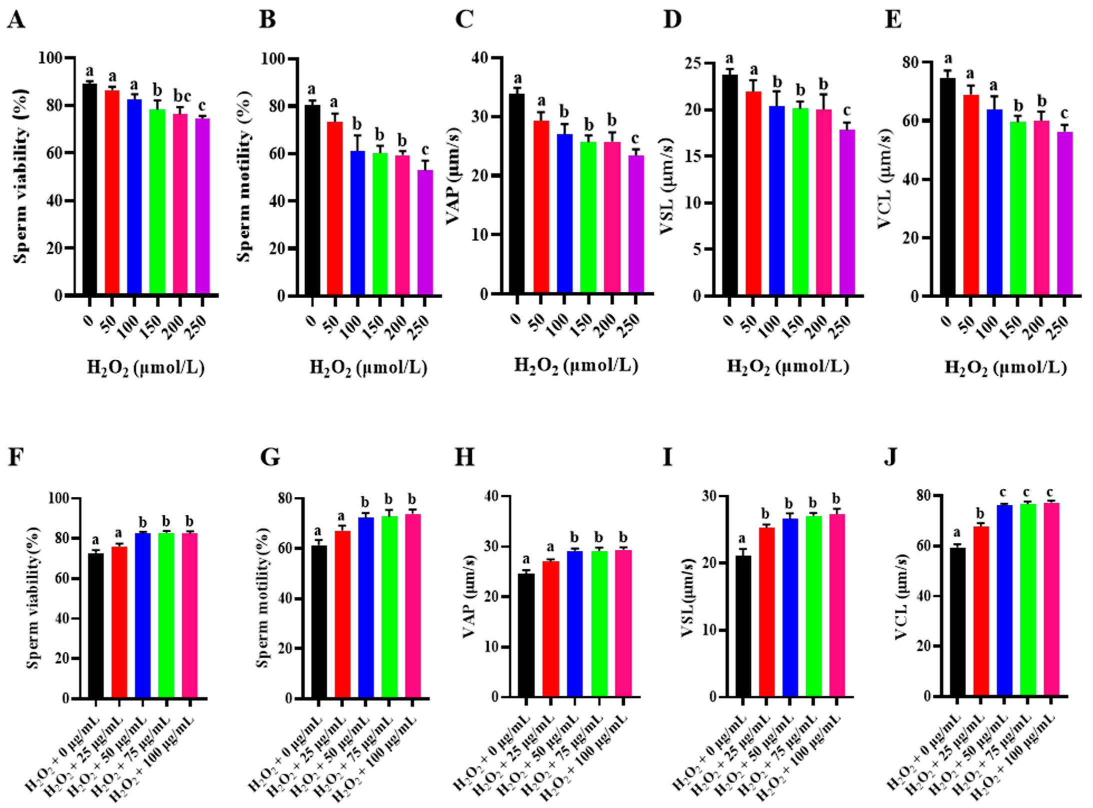

Figure. S1. Screening for suitable concentrations of H_2O_2 and NMN. (A) Sperm viability after treatment with H_2O_2 for 4 h. (B) Sperm motility. (C) VAP. (D) VSL. (E) VCL. (F) Sperm viability after treatment with NMN for 4 h. (G) Sperm motility. (H) VAP. (I) VSL. (J) VCL. The data are represented as mean ± SEM (n = 5), and different letter indicates significant difference ($P < 0.05$).

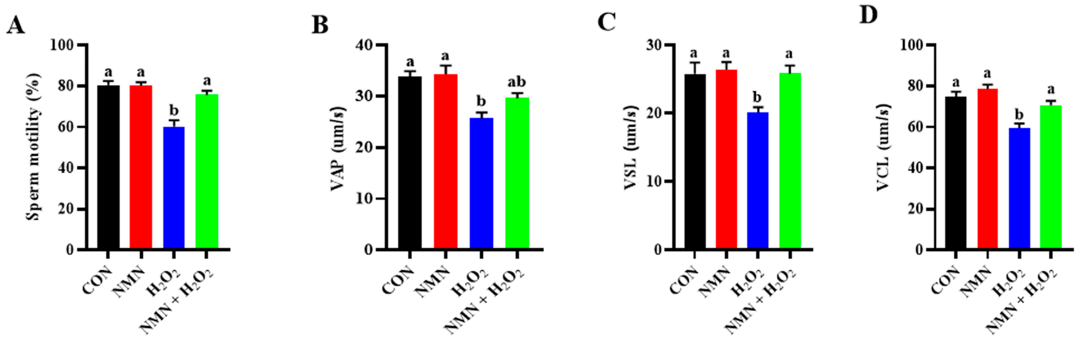

Figure. S2. NMN rescues sperm quality after H_2O_2 treatment. (A) Sperm motility after co-treatment with NMN and H_2O_2。 (B) VAP. (C) VSL. (D) VCL. The data are represented as

mean ± SEM (n = 5), and different letter indicates significant difference ($P < 0.05$).

Table S1

Composition and nutrient analysis of basal diet.

Ingredient	Content, %
Corn	37.48
Barley	15.22
wheat	14.81
Rice bran meal	10.17
Soybean meal	14.80
Soybean oil	2.50
L-lysine	0.40
Methionine	0.15
Threonine	0.25
Ground limestone	1.52
Monocalcium phosphate	1.21
Sodium chloride	0.49
Premix*	1.00
total	100
Nutrient, %	
Calculated NE, kcal kg^{-1}	2.30
Crude protein, %	16.20
Crude fat, %	3.86
Crude ash, %	6.74
Crude fiber, %	4.45
Ca, %	0.85
P, %	0.57

* Premix provided the following minerals per kilogram: VA 5800 IU, VD 3500 IU, VE 30 IU, VK 28.5 mg, VB 65.0 mg, VB1200 μg, biotin 0.25 mg, D-pantothenic acid 5.6 mg, nicotinic acid 10 mg, Cu 20 mg, Fe 180 mg, Zn 150 mg, Mn 60 mg, I 0.60 mg,

Se 0.60 mg, and Cr 0.25 mg.
